# Maternal Use of Antibiotics and the Risk of Childhood Febrile Seizures: A Danish Population-Based Cohort

**DOI:** 10.1371/journal.pone.0061148

**Published:** 2013-04-15

**Authors:** Jessica E. Miller, Lars Henning Pedersen, Mogens Vestergaard, Jørn Olsen

**Affiliations:** 1 Department of Epidemiology, School of Public Health, University of California Los Angeles, Los Angeles, California, United States of America; 2 Department of Obstetrics and Gynecology, Institute of Clinical Medicine, Aarhus University Hospital, Aarhus, Denmark; 3 Research Unit for General Practice and Department of General Practice, University of Aarhus, Aarhus, Denmark; 4 Department of Epidemiology, Institute of Public Health, Aarhus University, Aarhus, Denmark; Johns Hopkins University Bloomberg School of Public Health, United States of America

## Abstract

**Objective:**

In a large population-based cohort in Denmark to examine if maternal use of antibiotics during pregnancy, as a marker of infection, increases the risk of febrile seizures in childhood in a large population-based cohort in Denmark.

**Methods:**

All live-born singletons born in Denmark between January 1, 1996 and September 25, 2004 and who were alive on the 90^th^ day of life were identified from the Danish National Birth Registry. Diagnoses of febrile seizures were obtained from the Danish National Hospital Register and maternal use of antibiotics was obtained from the National Register of Medicinal Product Statistics. Hazard ratios (HR) and 95% confidence intervals (95% CI) were estimated by Cox proportional hazard regression models.

**Results:**

We followed 551,518 singletons for up to 5 years and identified a total of 21,779 children with a diagnosis of febrile seizures. Slightly increased hazard ratios were observed among most exposure groups when compared to the unexposed group, ex. HR 1.08 95% CI: 1.05–1.11 for use of any systemic antibiotic during pregnancy.

**Conclusion:**

We found weak associations between the use of pharmacologically different antibiotics during pregnancy and febrile seizures in early childhood which may indicate that some infections, or causes or effects of infections, during pregnancy could affect the fetal brain and induce susceptibility to febrile seizures.

## Introduction

Febrile seizures are the most common seizure disorder in children, affecting 2–5% of all children between 3 months and 5 years of age with a third having more than one episode. [1], [2] Children with febrile seizures may be at an increased risk of developing epilepsy. A population-based cohort found that less than 7 percent of children with febrile seizures went on to develop epilepsy by the age of 25; the risk was significantly higher for persons with a family history of epilepsy, cerebral palsy, or low Apgar score shortly after birth. [3] Genes and environmental factors operating in early life seem to play a causal role; more proximal causes of febrile seizures remain largely unknown. [4] Growing evidence suggests fetal exposures during gestation may contribute to neurological and mental disorders that manifest later in life [5] and several maternal infections during pregnancy have been associated with an increased risk of cerebral palsy [6], epilepsy [7], autism [8], mental retardation [9], [10] and schizophrenia [11] in the offspring. One possible mechanism is molecular mimicry, in which transplacentally-acquired antibodies to infectious agents with common epitopes of the developing nervous system may cross the fetus blood-nervous system barriers to cause neuropsychiatric disorders later in life. [12] Other mechanisms are possible but it remains unknown whether prenatal infections increase the susceptibility to seizures following exposure to fever.

We took interest in urinary tract infections because they are a common bacterial infection in pregnancy [13], [14] and have been associated with epilepsy in the offspring. [7] Symptomatic urinary tract infections are divided into lower tract (acute cystitis) and upper tract (acute pyelonephritis) infections. [15] In Denmark the medications used to treat uncomplicated acute cystitis are almost exclusively pivmecillinam, sulfamethizole, and nitrofurantoin; [16] these antibiotics are normally not used for other indications during pregnancy [17].

Prescription data, as a marker of infection, provides the ability to study non-hospitalized infections diagnosed by primary care doctors outside of hospitals or in outpatient clinical settings which may otherwise be excluded from hospital based studies. Our study examines the association between exposure to antibiotics during prenatal life and the risk of febrile seizures in a large population-based cohort study.

## Methods

### Ethics Statement

According to Danish laws, register-based studies do not need to obtain consent from individuals when personal identifiers have been encrypted and stored by a trusted third part (Statistic Denmark). The study was approved by the Danish Data Protection Agency (J.nr. 2009-41-3255).

### Study Population

Denmark has one of the world’s most comprehensive registration systems with extensive data on health and social conditions. [18] Residents in Denmark are assigned a unique personal identification number that enables linkage of individual information among all national registries. For our study, all live-born singletons born in Denmark between January 1, 1996 and September 25, 2004 and who were alive on the 90^th^ day of life and did not emigrate from Denmark were identified from the Danish National Birth Registry (N = 551,518). Of these, we identified 172,879 children born to women who took at least one type of antibiotic during pregnancy (exposed). The 378,639 children of women who did not take any antibiotics during pregnancy were considered unexposed (Table 1).

**Table 1:** Characteristics of eligible women with live-born singleton children, according to exposure status.

	Unexposed		Any systemic antibiotic			Cystitis Antibiotics		
	(n = 378,639 )		(n = 172,879)			(n = 68789)		
Characteristic	No.	%	No.	%	p-value*	No.	%	p-value*
**Sex**								
Girls	184565	(49)	84031	(49)	0.34	33527	(49)	0.98
Boys	194074	(51)	88848	(51)		35262	(51)	
Missing	0	(0)	0	(0)		0	(0)	
**Parity**								
0	168077	(44)	61961	(36)	<.001	31176	(45)	<.001
>1	200055	(53)	106734	(62)		35734	(52)	
Missing	10507	(3)	4184	(2)		1879	(3)	
**Gestational age (weeks)**								
<37	17707	(5)	8305	(5)	<.001	3471	(5)	<.001
37–41	328884	(87)	150330	(87)		59611	(87)	
> = 42	31978	(8)	14127	(8)		5645	(8)	
Missing	70	(0.02)	117	(0.07)		62	(0.09)	
**Maternal age (years)**								
<20	5585	(1)	3519	(2)	<.001	1795	(3)	<.001
20–24	48413	(13)	25392	(15)		12035	(18)	
25–29	139534	(37)	61290	(35)		25020	(36)	
30–34	129101	(34)	58011	(34)		20929	(30)	
> = 35	56006	(15)	24667	(14)		9010	(13)	
Missing	0	(0)	0	(0)		0	(0)	
**Smoking**								
No	288802	(76)	121892	(71)	<.001	49376	(72)	<.001
Quit after 1st trimester	4952	(1)	2165	(1)		986	(1)	
Smoked during pregnancy	69307	(18)	41489	(24)		15456	(22)	
Missing	15578	(4)	7333	(4)		2971	(4)	
**Cesarian section**								
No	281880	(74)	121512	(70)	<.001	50585	(74)	<.001
Yes	96249	(25)	50947	(29)		18097	(26)	
Missing	510	(0.13)	420	(0.24)		107	(0.16)	
**Apgar score 5 minutes**								
<10	27801	(7)	12622	(7)	<.001	5126	(7)	<.001
10	346637	(92)	158706	(92)		63010	(92)	
Missing	4201	(1)	1551	(1)		653	(1)	
**Parental Income** **								
Low	64499	(17)	33340	(19)	<.001	15110	(22)	<.001
Medium	169785	(45)	80197	(46)		30690	(45)	
High	135879	(36)	54944	(32)		21009	(31)	
Missing	8476	(2)	4398	(3)		1980	(3)	
**Congenital Malformation** ***								
No	368433	(97)	167982	(97)	<.01	66792	(97)	<.01
Yes	10206	(3)	4897	(3)		1997	(3)	
Missing	0	(0)	0	(0)		0	(0)	
**Birth weight (grams)**								
<2500	13253	(4)	6228	(4)	<.001	2646	(4)	<.001
>2500	361469	(95)	164949	(95)		65487	(95)	
Missing	3917	(1)	1702	(1)		656	(1)	
**Number of prescription redemptions**								
1	NA	NA	110766	(64)	<.001	36062	(52)	<.001
>1	NA	NA	62113	(36)		32727	(48)	

Note: Cystitis medications includes pivmecillinam, sulfamethizole, and nitrofurantoin.

* P-value comparing the ‘any systemic antibiotic’ and ‘cystitis antibiotics’ groups with the unexposed group using Pearson chi-square test for categorical factors.

** Parental income is based on parents’ combined annual income in the birth year of the child.

*** Includes ICD:10 codes Q00–Q79.

### Prescribed Antibiotics

Information on maternal use of antibiotics was obtained from the National Register of Medicinal Product Statistics, which contains individual-level information on all redeemed prescriptions in Denmark since 1994 except on drugs sold without a prescription or drugs for inpatient use only. Drugs are coded according to the WHO Anatomical Therapeutic Chemical (ATC) classification system. ATC codes and date of sale for each prescription are stored in the database when redeemed. The ATC system categorizes medicinal substances at five different levels according to the organ or system on which they act and their chemical, pharmacological and therapeutic properties. Antibiotics for the study were defined with ATC codes ‘J01’ (any systemic antibacterial), ‘J01C’ (beta-lactam antibacterials, penicillins), ‘J01E’ (sulfonamides and trimethoprim), ‘J01F’ (macrolides, lincosamides and streptogramins), ‘J01X’ (other antibacterials), ‘J01CE02’ (phenoxymethylpenicillin (penicillin V)), ‘J01FA01’ (erythromycin), ‘J01CA08’ (pivmecillinam), ‘J01EB02’ (sulfamethizole), and ‘J01XE01’ (nitrofurantoin). Mothers were classified as being exposed during pregnancy if they had redeemed a prescription for an aforementioned medication with the date of sale between the start of pregnancy and the date of birth of the child. Mothers were classified as being unexposed to antibiotics during pregnancy if they did not have a redeemed prescription for any systemic antibiotic (ATC: J01) between the start of pregnancy and the date of birth of the child. The initiation of pregnancy (first day in the last menstrual period) was calculated by subtracting gestational age in days from the day of birth.

### Febrile Seizures

We obtained information on first event of febrile seizures from the Danish National Hospital Register, [19] which contains information on discharge diagnoses for all patients from Danish hospitals and outpatient clinics. Diagnostic information is based on the Danish version of the *International Classification of Diseases,* 10^th^ Revision (ICD-10) from 1994 onward and reported to the register after each hospital visit. [20] Cohort members were identified as having febrile seizures if they were between the ages of 3 months and 5 years and if they had been hospitalized or had been in outpatient care because of a primary, secondary or underlying diagnosis of febrile seizures (ICD-10 code R56.0) and no previous history of epilepsy (ICD-10:G40–G41), intracerebral infection (ICD-10: G00–G09), or cerebral palsy (ICD-10: G80–G83). Time of onset of febrile seizures was defined as the first day of contact with the hospital when patients were hospitalized or in outpatient care with the first discharge diagnosis of febrile seizures. All treatments in Danish hospitals are free of charge for residents.

### Potential Confounders, Intermediate Factors, and Covariates

Information on maternal age, gestational age at birth, parity, Apgar score at 5 minutes, sex, smoking status during pregnancy, method of delivery, and birth year was obtained from the Danish Medical Birth Registry. [21] Information on gestational age at birth is usually estimated from ultrasound measures during early pregnancy but in the few cases where ultrasound measures were not taken, the last menstrual date was used in the time period of this study.

Information on parental income was obtained from the Fertility Database which contains annually collected education, employment, and family/housing information for people of fertile age in Denmark. [22] Parental income was defined as the combined income of the mother and father during the child’s birth year. If information on income for both parents was missing, data from the previous calendar year were used if available.

Information on congenital malformations was obtained from the Danish National Hospital Register (ICD-10: Q00–Q79). ICD-10 codes for “other congenital malformations” and “chromosomal abnormalities, not elsewhere classified” (Q80–Q99) were not included in our definition of congenital malformation.

### Statistical Analysis

We modeled the risk of febrile seizures in the children over time in ten different maternal antibiotic exposure groups. All children were followed from the 90^th^ day of life until the first diagnosis of febrile seizures, death, until they reached 5 years of age, or December 31, 2007, whichever occurred first. Hazard ratios and 95% confidence intervals (CI) were estimated by Cox proportional hazard regression models with person-years as the time-to event variable. Causal diagrams [23] (directed acyclic graphs) provide a method for evaluation of confounders and mediators and were used to guide the selection of potential confounders to be controlled for in our final models. All hazard ratios were adjusted for maternal age, parental income, smoking status during pregnancy, and birth year. The model was also run with the additional adjustment for the maternal conditions of chorioamnionitis (ICD-10: O41.1), gestational diabetes mellitus (ICD-10: O24.4 and O24.9), and any other prescribed maternal medication besides antibiotics during pregnancy (ATC code other than J01). The validity of the proportional hazards assumption of the Cox models was evaluated for time-dependent covariates; the assumption was not rejected for any variables. Missing values were included as distinct categories for smoking and income (<5%) to maintain the sample size. Robust standard errors adjusted for dependency between multiple pregnancies of women during the study period (n = 146,399 mothers had multiple pregnancies). Analyses were performed using PROC PHREG in SAS version 9.1.

## Results

We followed 551,518 singletons for up to 5 years of age (median = 4.75 years) and identified a total of 21,779 children with a diagnosis of febrile seizures in the full cohort. Women who were exposed to cystitis antibiotics during the study period were on average slightly younger than unexposed women, with a larger percentage of women aged 20–24 years, and had slightly lower income (Table 1). A larger percentage of unexposed women and women exposed to cystitis antibiotics had on average lower parity (no previous births) compared to women exposed to antibiotics other than cystitis antibiotics. While the percentage of women exposed to antibiotics over the birth years is fairly consistent, the percentage exposed to pivmecillinam and nitrofurantoin gradually increased over time (data not shown). All other characteristics appear comparable between the exposed and unexposed groups. The majority of sulfonamides and trimethoprim and other antibacterials antibiotics were comprised of the specified cystitis antibiotics, 99% sulfamethizole (monotherapy) and 98% nitrofurantoin, respectively.

We found slightly higher risks of febrile seizures among children born to mothers in all exposure groups except for antibiotics categorized as macrolides, lincosamides and streptogramins when compared to children born to unexposed mothers. The hazard ratios for the different exposure groups ranged from 1.06 to 1.16 (Table 2). Hazard ratios were similar for boys and girls (Table S1). Controlling for the maternal conditions of chorioamnionitis, gestational diabetes mellitus, and any other prescribed medication did not change the overall hazard ratios (data not shown).

**Table 2:** Unadjusted and adjusted hazard ratios for risk of febrile seizures, according to antibiotic exposure.

ANTIBIOTIC	N (Cases)	Incidence rate per 100,000 person years	Unadjusted HR	Adjusted HR (95% CI)
Unexposed	378639 (14550)	862	reference	reference
Any systemic antibiotic	172879 (7229)	942	1.09	1.08 (1.05–1.11)
Beta-lactam antibacterials, penicillins	135925 (5656)	939	1.09	1.07 (1.04–1.11)
* Penicillin V*	79063 (3242)	922	1.07	1.06 (1.02–1.10)
* Pivmecillinam* *	34596 (1508)	999	1.14	1.12 (1.06–1.18)
Sulfonamides and Trimethoprim	37991 (1662)	987	1.14	1.12 (1.06–1.18)
Sulfamethizole*	37648 (1649)	988	1.14	1.12 (1.06–1.18)
Macrolides, Lincosamides and Streptogramins	20451 (844)	923	1.07	1.06 (0.98–1.13)
* Erythromycin*	15886 (638)	896	1.05	1.03 (0.95–1.11)
Other antibacterials	7070 (315)	1022	1.17	1.15 (1.03–1.28)
* Nitrofurantoin* *	6926 (312)	1034	1.18	1.16 (1.04–1.29)

Adjusted models were adjusted for maternal age, SES, smoking status during pregnancy, birth year.

* Medications commonly used for treatment of cystitis in Denmark.

Additional analysis was done in a subgroup of children with no apparent markers of serious health problems at the time of birth, defined as children born at term (37–41 weeks), with a birth weight >2500 g, no major congenital malformations, and an Apgar score at 5 minutes ≥10. Rather similar results to the larger study population were seen for this subgroup (Table 3). In a separate analysis these variables were included in the model and the results remained the same (data not shown). Analysis in first born children presented statistically significant associations in all exposure groups, except in children born to mothers exposed to erythromycin and other antibacterials, when compared to children born to unexposed mothers (data not shown).

**Table 3:** Hazard ratios for risk of febrile for children born at term with a birth weight >2500 g, no congenital malformations, and Apgar score at 5 minutes >10, in study population.

ANTIBIOTIC	N (Cases)	Incidence rate per 100,000 person years	Adjusted HR (95% CI)
Unexposed	291122 (10942)	843	reference
Any systemic antibiotic	133154 (5438)	920	1.08 (1.04–1.11)
Beta-lactam antibacterials, penicillins	104894 (4262)	916	1.07 (1.03–1.11)
* Penicillin V*	61346 (2472)	905	1.06 (1.02–1.11)
* Pivmecillinam* *	26585 (1142)	986	1.13 (1.06–1.20)
Sulfonamides and Trimethoprim	28880 (1229)	960	1.11 (1.05–1.18)
Sulfamethizole*	28613 (1219)	961	1.11 (1.05–1.18)
Macrolides, Lincosamides and Streptogramins	15725 (640)	909	1.06 (0.98–1.15)
* Erythromycin*	12227 (470)	856	1.00 (0.92–1.10)
Other antibacterials	5451 (237)	998	1.15 (1.01–1.31)
* Nitrofurantoin* *	5354 (235)	1008	1.16 (1.02–1.32)

Adjusted for maternal age, SES, smoking status during pregnancy, birth year.

* Medications commonly used for treatment of cystitis in Denmark.

We considered the number of redeemed prescriptions by the mothers as an indicator of actual exposure to infection and length of exposure time, assuming more than one redeemed prescription suggests either recurrent infections, resistance to the first used antibiotics, or an overall longer period of time exposed to the underlying infection. Stratum-specific estimates among those who had either one or more than one redeemed prescription for the antibiotics during pregnancy are presented in Table 4. Compared to children born to unexposed mothers, children born to mothers with more than one redeemed prescription had statistically significant associations in all exposure groups except for mothers exposed to macrolides, lincosamides and streptogramins.

**Table 4:** Hazard ratios for risk of febrile seizures in the children, by number of redeemed prescriptions during pregnancy, in study population.

	1 redemption				>1 redemption		
ANTIBIOTIC	N (Cases)	Incidence rate per 100,000 person years	Adjusted HR (95% CI)	N (Cases)		Incidence rate per 100,000 person years	Adjusted HR (95% CI)
Unexposed	378639 (14550)	862	ref	378639 (14550)		862	ref
Any systemic antibiotic	110766 (4558)	927	1.06 (1.03–1.09)	62113 (2671)		971	1.11 (1.06–1.15)
Beta-lactam antibacterials, penicillins	80845 (3294)	918	1.05 (1.01–1.09)	55080 (2362)		968	1.10 (1.06–1.15)
* Penicillin V*	45823 (1826)	895	1.03 (0.98–1.08)	33240 (1416)		959	1.10 (1.04–1.16)
* Pivmecillinam* *	15972 (694)	997	1.12 (1.04–1.21)	18624 (814)		1002	1.12 (1.04–1.20)
Sulfonamides and Trimethoprim	18118 (760)	942	1.07 (0.99–1.16)	19873 (902)		1028	1.16 (1.08–1.24)
Sulfamethizole*	18011 (758)	946	1.08 (1.00–1.16)	19637 (891)		1028	1.16 (1.08–1.24)
Macrolides, Lincosamides and Streptogramins	8885 (380)	954	1.09 (0.99–1.21)	11566 (464)		899	1.02 (0.93–1.12)
* Erythromycin*	6608 (274)	923	1.06 (0.94–1.19)	9278 (364)		876	1.00 (0.90–1.11)
Other antibacterials	2113 (94)	1023	1.15 (0.94–1.40)	4957 (221)		1022	1.15 (1.00–1.31)
* Nitrofurantoin* *	2079 (94)	1041	1.17 (0.95–1.43)	4847 (218)		1032	1.16 (1.01–1.32)

Adjusted for maternal age, SES, smoking status during pregnancy, birth year.

* Medications commonly used for treatment of cystitis in Denmark.

## Discussion

In this population-based cohort we observed statistically significant but weak associations between maternal use of several different types of antibiotics and the onset of febrile seizures in children both before and after adjusting for potential confounding factors (Table 2). Such weak associations may be due to uncontrolled confounding but may also reflect strong associations related to specific infections. To adjust for risk factors associated with poor health indicators at birth, we performed analyses in a subgroup of children with no evident signs of serious health problems, and the associations still held. Additionally, we looked at the association in first born children to eliminate bias related to the outcome in a previous pregnancy, and the associations still held. The hazard ratios we observed correspond to small increases in absolute rates among the unexposed and exposed groups, which may not be of much importance, unless it indicates that some infections may alter the susceptibility to febrile seizures or infections during the intrauterine time period. The mechanism could be related to mimicry mechanisms, by affecting the immune system, or by other mechanisms. We are unaware of any studies researching maternal antibiotics as a marker of maternal infection and the risk of febrile seizures in childhood. We encourage others to study specific infections.

Whether the associations we observed were caused by confounding, underlying infection, or treatment is difficult to say but these drugs are not believed to be neurotoxic and we were able to adjust for several potential confounders. Observing an increased risk for the different cystitis antibiotics, regardless of chemical component, supports and strengthens the argument that the prenatal infection may be the underlying cause of the association. If a direct effect of the antibiotics was responsible for the increased risk of febrile seizures we would expect to see more variation in hazard ratios between the different pharmacokinetic and –dynamic antibiotics. Though associations were found for systemic antibiotics overall and for antibiotics not specifically used to treat cystitis, these associations are more difficult to interpret given the heterogeneous underlying diseases.

The validity of using antibiotics as a marker of infection should be considered. Infections are common disorders and some women among our unexposed group may have had the disease either untreated or treated with non-prescription medication or with medication that was bought outside the pregnancy time period. It is possible some women may have bought medication without having the disease at that time, for example, as a prophylactic treatment. For these reasons we considered the number of redeemed prescriptions as a possible indicator of actual use of antibiotics and exposure to infection during pregnancy. Women who redeemed medications more than once probably had the disease or recurrent infection and we observed an increase in estimates for penicillin, sulfonamides and trimethoprim overall, and sulfamethizole when more than one prescription was redeemed. These estimates do not necessarily represent a dose-response where >1 redeemed prescription would suggest a more severe underlying disease, but may be more indicative of a dose-duration and longer exposure time.

The trimester in which antibiotics were redeemed may not equal the initiation of infection. In our study, the actual onset of infection may differ considerably from the time at which antibiotics were redeemed, for example redemptions during the 2^nd^ or 3^rd^ trimester may be for infections that originated during the first trimester, but did not present until later. Thus, assessing “the timing of infection” based on “the date of redemption” will be an imprecise measure of timing of exposure.

While antibiotics may be a marker of infection, at the same time they are a treatment that attempts to shorten the time and severity of infections and therefore may prevent or mask an association between prenatal infections and febrile seizures. In this case our estimates could be severely biased towards the null, especially if any specific microorganisms play a role. Infections have been associated with abnormal fetal brain development and adverse outcomes such as cerebral palsy [6], mental retardation [9], [10], epilepsy [7] and schizophrenia [11], yet the mechanisms underlying the associations are not known. A possible causal mechanism may be in part due to the maternal inflammatory response where dysregulation of the normal expression of cytokines in the fetal brain may affect neurodevelopmental processes. [24] In animal studies, cytokine releasing treatments during pregnancy have been associated with fetal brain injury.[25]–[27] Another hypothesized mechanism is that of epitope molecular mimicry of transplacentally-acquired maternal antibodies with the developing fetal nervous system that can cross the fetal blood-brain barriers. For example, polysialic acid, common to particular components of the nervous system, is found in large quantities in early fetal/infant development. Escherichia coli is the primary pathogen of maternal urinary tract infections and the polysialic acid found in the infectious agent E. coli K1 is identical to the nervous system associated polysialic acid molecules. [28] However, these mechanisms are currently not well documented. Fever has been suggested as being a reproductive hazard in animal studies [29] but is an unlikely explanation for our results. Cystitis usually does not cause fever but fever may play a role for other infections. [30].

Our study has several strengths including the use of population-based registries with almost no loss to follow up, well-documented redeemed prescription data, and reliable hospitalization information. A validation study by Vestergaard et al. concluded that registration of febrile seizures in the National Hospital Register is relatively complete and valid with a completeness found to be 72% (95% CI: 66.3–76.4) and a predictive value of a positive registration of 93% (95% CI: 88.8–95.7). [2] However the register does not contain information on type of febrile seizures (focal or generalized), duration of febrile seizure, or recurrence within 24 hours, so cases cannot be categorized as simple or complex. [31] Since registration of febrile seizures is done independently of data on redeemed prescriptions differential misclassification is not of concern. Although some seizures may herald other progressive neurological conditions and be misdiagnosed as febrile seizures, the high positive predictive value 93% (95% CI: 88.8–95.7) of the hospital register along with the estimated less than 7 percent of children with febrile seizures who develop epilepsy later in life [3] would suggest only a very small percent of misdiagnoses.

We controlled for maternal smoking during pregnancy but not for other lifestyle factors, such as alcohol, diet, or coffee consumption. However, based on a study by Vestergaard et al., [32] prenatal exposure to cigarette smoke only slightly increased risk for febrile seizures in children, but no associations were found for exposure to alcohol or coffee. Our results showed a similar increase in risk for febrile seizures if mothers smoked during pregnancy, compared to non-smokers, which was consistent across all studied medications. We observed a lower parental income level only marginally increased the risk of febrile seizures when compared to a middle parental income level. Our measure of parental income was limited to the parents’ combined annual income and may not have adequately controlled for effects of social confounding. We addressed possible confounding associated with multiple children in the family through our subgroup analysis in first born children; however, there may be other variables which we did not consider. We controlled for certain maternal conditions in a separate model but this did not alter our effect estimates. While the etiology of febrile seizures may have a genetic component, we did not have complete data on parental history of febrile seizures and were unable to adjust for this variable. Additional adjustments for unknown or unmeasured social factors may explain some of the increases in estimates.

## Conclusions

We found weak associations between the redemption of certain antibiotics during pregnancy and febrile seizures in early childhood. We found comparable associations for pharmacologically different antibiotics which suggest an association with the risk of infection itself rather than the exposure to antibiotics. Further research on the biomarkers of infection and the specific mechanisms of how maternal infection may affect the developing fetus is encouraged.

## Supporting Information

Table S1 **Hazard ratios (and 95% confidence intervals) for the risk of febrile seizures in children, by gender, whose mothers had a redeemed prescription for a systemic antibiotic during pregnancy compared to children whose mothers did not have a redeemed prescription for an antibiotic during pregnancy.**
(DOCX)
