# Supplementary material for: Maternal Use of Antibiotics and the Risk of Childhood Febrile Seizures: A Danish Population-Based Cohort
Source: PLoS One. 2013 Apr 15;8(4):e61148. doi: 10.1371/journal.pone.0061148 (PMC3627381; doi:10.1371/journal.pone.0061148)
Supplement: Table S1 — Hazard ratios (and 95% confidence intervals) for the risk of febrile seizures in children, by gender, whose mothers had a redeemed prescription for a systemic antibiotic during pregnancy compared to children whose mothers did not have a redeemed prescription for an antibiotic during pregnancy. (DOCX) [file pone.0061148.s001.docx]

|  | | Female | | | Male | | |
| --- | --- | --- | --- | --- | --- | --- | --- |
| ANTIBIOTIC | | N | Cases | Adjusted  HR (95%CI) | N | Cases | Adjusted  HR (95%CI) |
| Unexposed | | 184565 | 6407 | reference | 194074 | 8143 | reference |
| Any systemic antibiotic | | 84031 | 3213 | 1.09 (1.05 - 1.14) | 88848 | 4016 | 1.07 (1.03 - 1.11) |
| Beta-lactam antibacterials, penicillins | | 66119 | 2534 | 1.09 (1.04 - 1.15) | 69806 | 3122 | 1.06 (1.01 - 1.10) |
|  | *Penicillin V* | 38435 | 1442 | 1.07 (1.01 - 1.14) | 40628 | 1800 | 1.05 (0.99 - 1.10) |
|  | *Pivmecillinam** | 16913 | 718 | 1.21 (1.12 - 1.30) | 17683 | 790 | 1.05 (0.97 - 1.13) |
| Sulfonamides and Trimethoprim | | 18368 | 724 | 1.12 (1.04 - 1.21) | 19623 | 938 | 1.12 (1.04 - 1.19) |
|  | *Sulfamethizole** | 18205 | 715 | 1.11 (1.03 - 1.20) | 19443 | 934 | 1.12 (1.05 - 1.20) |
| Macrolides, Lincosamides and Streptogramins | | 9899 | 345 | 0.98 (0.88 - 1.09) | 10552 | 499 | 1.11 (1.01 - 1.21) |
|  | *Erythromycin* | 7664 | 266 | 0.98 (0.87 - 1.11) | 8222 | 372 | 1.06 (0.96 - 1.18) |
| Other antibacterials | | 3526 | 148 | 1.19 (1.02 - 1.41) | 3544 | 167 | 1.11 (0.95 - 1.29) |
|  | *Nitrofurantoin** | 3454 | 147 | 1.21 (1.03 - 1.43) | 3472 | 165 | 1.12 (0.96 - 1.31) |
| Adjusted for maternal age, SES, smoking status during pregnancy, birth year | | | | | | | |
| * Medications commonly used for treatment of cystitis in Denmark | | | | | | | |

Table S1 – Hazard ratios for risk of febrile seizures in the children by gender, in study population
